# More than a rigid framework: molecular design using secondary structure element information

**DOI:** 10.1186/1758-2946-5-S1-P45

**Published:** 2013-03-22

**Authors:** Oliver Koch

**Affiliations:** 1 Institute of Pharmacy, Eberhard-Karls-University Tübingen, Germany

## 

The structure-based design of small molecule modulators of protein-ligand binding and protein-protein interaction is a key component in drug discovery. The underlying protein interactions can be regarded based on structural similarity of the involved secondary structure elements [1].

The most prominent example is the protein fold of a protein domain that is more conserved than the amino acid sequence and proteins with similar fold but dissimilar sequence and function can bind similar ligands. Similar ligand binding can also occur between different proteins that have a similar spatial arrangement of secondary structure elements around the ligand binding site ("ligand-sensing cores") independent from the overall fold [2]. Analogous to this, similar protein-protein interfaces can occur within different protein folds showing different function, since the structural space of protein interfaces is degenerated and is represented by roughly 1000 distinct interfaces [3].

These similarities in otherwise unrelated proteins can be useful in the design of protein function modulators. The successful applications in molecular design described in literature using predicted polypharmacology in protein-ligand binding will be shown and the analogy in the design of protein-protein interaction inhibitors and the potential of polypharmacology prediction will be discussed.

## References

[B1] Koch O Use of secondary structure element information in drug design: polypharmacology and conserved motifs in protein-ligand binding and protein-protein interfaces Future Med Chem 2011 3 699 708 10.4155/fmc.11.26 21554076

[B2] Koch MA Waldmann H Protein structure similarity clustering and natural product structure as guiding principles in drug discovery Drug Discov Today 2005 10 471 483 10.1016/S1359-6446(05)03419-7 15809193

[B3] Gao M Skolnick J Structural space of protein-protein interfaces is degenerate, close to complete, and highly connected Proc Natl Acad Sci USA 2010 107 22517 22522 10.1073/pnas.1012820107 21149688 PMC3012513

